# Distribution and characteristics of rearranged hopanes in the black shale of the Chang 9 member, the Upper Triassic Yanchang Formation in the Ansai area, Ordos Basin, North China

**DOI:** 10.1371/journal.pone.0337076

**Published:** 2025-12-01

**Authors:** Han Yue, Yan Liu, Rongxi Li, Xuefeng Liu

**Affiliations:** 1 School of Earth Science and Resources, Chang’an University, Xi’an, PR China; 2 School of Science, Chang’an University, Xi’an, PR China; 3 Xingzichuan Oil Production Plant of Yanchang Oil Field Co., Ltd, Yan’an, PR China; Makerere University College of Natural Sciences, UGANDA

## Abstract

Shale samples from source rocks of the Upper Triassic Yanchang Formation (Chang 9 member) in the Ansai area, Ordos Basin, North China, were analyzed using gas chromatography – mass spectrometry (GC-MS) to investigate the distribution, abundance, and enrichment mechanisms of rearranged hopanes. Four rearranged hopane series were detected, with all four present simultaneously in individual samples. Analysis of the C₃₀ hopane series (regular C₃₀H, diahopane C₃₀D, and neohopane C₃₀E) using a ternary diagram revealed a distinct linear trend, demonstrating a systematic, inverse relationship between the abundance of regular hopane and the combined abundance of its rearranged counterparts. These results provide strong evidence that C₃₀D and C₃₀E in the Chang 9 shales are diagenetic products derived from C₃₀H, sharing a common biological precursor. Both diasteranes and regular steranes with the ββ configuration were correlated positively in abundance with rearranged hopanes, further supporting a common origin linked to specific organism assemblages rather than widespread organisms. Samples deposited under highly saline, suboxic sedimentary environments displayed relatively high abundances of rearranged hopanes, indicating the critical role of depositional conditions in their enrichment. Multi-proxy analysis revealed a complex, non-linear control of thermal maturity on rearranged hopane abundance. The C₃₀ Rearranged Hopane Index showed statistically significant positive correlations with multiple maturity parameters (including sterane and hopane isomerization ratios), indicating maturity as a primary driver in the early oil window. However, this trend diverged at higher maturity levels, suggesting that other factors, such as the catalytic activity of the mineral matrix, become dominant. Our findings establish a robust biomarker-based framework for interpreting oil-source correlations and informing petroleum exploration in the Ordos Basin, particularly for the Chang 9 member source rocks.

## 1 Introduction

Regular 17α(H), 21β(H) hopanes, with carbon numbers ranging from C_27_ to C_35_, are abundant in sediments and crude oils from many basins worldwide [1–7]. Rearranged hopanes are a class of biomarker compounds with the same carbon skeleton as regular hopanes but differing substitution patterns along methyl side chain. Four series of rearranged hopanes have been reported and identified in sediments and crude oils, including 17α(H)-diahopanes (D series), early-eluting series (E series), 18α(H)-neohopanes (Ts series), and 21-methyl-28-norhopanes (Nsp series). 18α(H)-22,29,30-Trisnorneohopane (Ts) of the Ts-series was the first compound to be structurally identified by X-ray crystallography, having a rearranged methyl group at C-17 [8,9]. The other known compound of the Ts series identified was 18α(H)-30-neohopane (C_29_Ts) using nuclear magnetic resonance (NMR) techniques [10]. Moldowan et al. also discovered a novel rearranged hopane series, the D-series, with carbon numbers C_29_–C_34_, using gas chromatography-mass spectrometry (GC-MS) [10]. The early-eluting rearranged hopane series (E-series) has been observed in lacustrine oils and detected using ion monitoring [2,11]. The E-series, C_27_E and C_29_E–C_35_E, are characterized by elution about two carbon numbers earlier than the corresponding regular hopanes of the same carbon number [3]. Huang et al. first reported the C_29_ 28-nor-spergulane (C_29_Nsp), a member of the Nsp series, in the western Pearl River Basin off the coast of South China [12]. Subsequently, Nytoft et al. identified the Nsp series ranging from C_29_ to at least C_34_ using NMR techniques [13].

Although the origin and geochemical significance of rearranged hopanes have been widely studied since their identification, their formation mechanisms remain controversial. 17α(H)-Diahopanes, first observed in terrigenous oils and coals, are considered terrestrial biomarkers [14]. Killops and Howell postulated that rearranged hopanes terrigenous organic matter that was bacterially reworked [2]. It was then suggested that their biological precursors were bacterial in origin because of their isotopic similarity to regular hopanes [10]. However, Zhang et al. suggested that some specific algae (e.g., rhodophytes) may also have been sources of rearranged hopanes [4]. Crucially, the conversion from biological precursors to rearranged hopanes is now widely understood to be a diagenetic, acid-catalyzed process rather than a direct inheritance from organisms [15–17]. The catalytic effect of acidic clay minerals in the sediment matrix is considered a primary driver of this molecular rearrangement. For instance, studies on Chinese lacustrine and coal measure rocks have demonstrated that acidic clays, such as kaolinite and smectite, facilitate the formation of rearranged hopanes, with their abundances sometimes showing a positive correlation with clay content [17]. However, this relationship is not always straightforward; other factors within the depositional environment, lithology, redox and/or pH conditions, and rock fabrics have been considered to be the most important factors influencing the relative abundance of rearranged hopanes [3,14]. The potential applications of rearranged hopanes as geological markers in oils and sediments are becoming increasingly important, including oil maturity assessment, oil family classification, and oil-source correlation studies [3,6,9,12,18,19]. Consequently, various ratios based on the increasing thermodynamic stability of the rearranged products with thermal maturation have been developed as widely used maturity indicators. These include the classic Ts/(Ts + Tm) and C₂₉Ts/(C₂₉Ts + C₂₉H) ratios, as well as ratios involving C₃₀ 17α(H)-diahopane [5,20–22].

Despite these advances, the interplay between controlling factors (e.g., organic matter source, clay catalysis, depositional environment, and thermal maturity) can vary significantly across geological settings, leading to regional variations in rearranged hopane distributions. The Chang 9 member in the Ordos Basin is a critical source rock, yet the distribution patterns and controlling mechanisms of rearranged hopanes within it have not been systematically investigated. A lack of understanding of these controls has hindered precise oil-source rock correlations in the study area, particularly for tight oil exploration where migration distances are short. Therefore, this study: (1) systematically characterizes the distribution of four series of rearranged hopanes in the Chang 9 source rocks from the Ansai area; (2) investigates the potential roles of organic matter input, depositional environment, and thermal maturity in controlling their abundances; and (3) establishes a more robust biomarker framework to support petroleum exploration in the Ordos Basin.

## 2 Geological background

**Fig 1:**
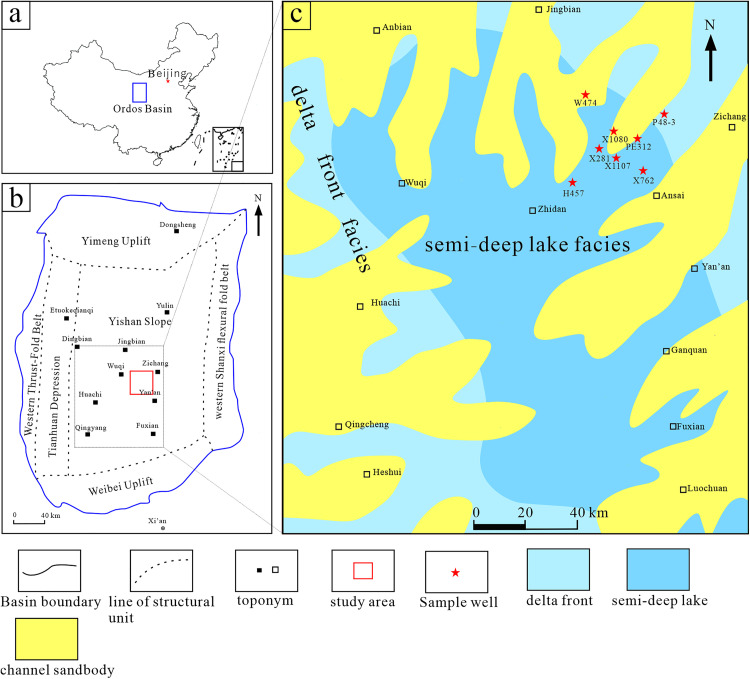
Location, structural framework, and sedimentary facies distribution of the study area in the Ordos Basin, China. **(a)** Location of Ordos basin, China. **(b)** Structural division of the Ordos Basin and the studied area. **(c)** Sedimentary facies of the Chang 9 section, Upper Triassic Yanchang Formation. The base map for panel (a) was generated using QGIS software with public domain data (Projection: WGS84 (EPSG:4326)) from Natural Earth (www.naturalearthdata.com). The geological maps in panels (b) and (c) were compiled based on previous studies and internal data.

The Ordos Basin is the second-largest sedimentary basin in China and the largest oil and gas production base (Fig 1a). The Mesozoic strata of the basin host substantial quantities of oil and gas resources, with reservoirs exhibiting low-to-ultralow porosity and permeability. The Upper Triassic Yanchang Formation represents a series of fluvial-lacustrine terrestrial clastic sedimentary systems deposited during a period of continuous depression and stable subsidence within the basin. The Yanchang Formation has been subdivided into 10 oil-bearing formations, designated from top to bottom as Chang 1 to Chang 10, based on sedimentary cycles and lithological characteristics [21]. These formations are regarded as the principal oil-bearing layers [22]. Previous studies have identified the Chang 7 member as the primary source rock in the Ordos Basin due to its extensive distribution and high-grade lacustrine source rocks [22,23]. Recently, shale source rocks have been identified in the Chang 9 member [4,24]. Additionally, the Chang 8–10 reservoirs adjacent to the Chang 9 source rock have demonstrated promising oil and gas potential. Furthermore, industrial oil flow has been successfully obtained [25], suggesting that the Chang 9 source rock may play a critical role in oil and gas generation.

The sedimentary period of the Chang 9 member represents the initial phase of lake basin formation, characterized by the accumulation of delta-lake facies deposits. The deposition of these sediments corresponds to the first lake invasion during the Late Triassic. At the end of the Chang 9 period, the lake basin expanded to its maximum extent along the Wuqi–Zhidan–Ganquan–Huangling section, resulting in the deposition of a narrow and semi-deep lake sediment commonly known as the Lijiapan Shale [26]. Previous studies have demonstrated that the Chang 9 shale is an effective source rock with proven hydrocarbon generation and expulsion potential [24]. The organic matter content of the Chang 9 shales is relatively high, ranging from 0.53% to 10.5% and averaging at approximately 5.0%. The organic matter is derived from both lower aquatic organisms and higher terrestrial plants. The organic matter types are predominantly Types I and II_1_, which are in the mature stage. The hydrocarbon generation and transformation rates of source rocks in the Zhidan–Ansai region are high, indicating a significant capacity for hydrocarbon generation and migration [27,28].

## 3 Materials and methods

### 3.1 Materials

Eight core samples were collected from the Chang 9 member of the Yanchang Formation (Fig 1c). Following common practice [29,30], we used TOC > 1 wt% to designate black shale. Accordingly, our dataset included both black shales (TOC > 1 wt. %) and lower‑TOC shales (TOC < 1 wt. %). TOC ranged from 0.12 to 2.0 wt% (Table 1) and average extractability was 0.22 mg/g. All samples contained Type I–II kerogens and were within the peak oil window of thermal maturity based on Rock‑Eval parameters.

**Table 1:** *n*-alkanes and acyclic isoprenoids parameters of the Chang 9 shales.

samples	CPI	OE	*Σn*-C_21_-/Σ*n*-C_22_+	(*n*-C_21_ + *n*-C_22_)/(*n*-C_28_ + *n*-C_29_)	TAR	Pr/*n*-C_17_	Ph/*n*-C_18_	Pr/Ph
X1080	1.16	1.03	0.61	1.38	1.06	0.41	0.37	0.76
X1107	1.41	1.5	0.76	0.98	0.87	0.80	0.39	2.1
H457	1.07	1.02	1.03	2.0	0.57	0.11	0.06	1.5
PE312	1.12	0.98	0.82	1.8	0.72	0.31	0.09	3.4
X762	1.08	1.00	1.22	1.8	0.51	0.19	0.09	2.1
W474	1.08	1.00	1.00	1.7	0.64	0.25	0.11	2.1
P48-3	1.05	1.08	2.0	2.6	0.35	0.13	0.09	1.6
X281	1.09	1.07	1.6	2.1	0.48	0.21	0.10	2.1

CPI: Carbon preference index over the *n*-C_24_ to *n*-C_32_ range; OEP: Odd-over-even preference ((*n*-C_21_ + 6 × *n*-C_23_ + *n*-C_25_)/(4 × *n*-C_22_ + 4 × *n*-C_24_)). TAR: Terrigenous/aquatic ratio (*n*-C_27_ + *n*-C_29_ + *n*-C_31_)/(*n*-C_15_ + *n*-C_17_ + *n*-C_19_); Pr: Pristane; Ph: Phytane.

**Permits and approvals:** No field sampling was conducted for this study. All samples were obtained by subsampling archived drilling cores owned and curated by Shaanxi Yanchang Petroleum (Group) Co., Ltd. (Xingzichuan Oil Production Plant), at the core repository in Yan’an, Shaanxi Province, China. Access to the cores and permission to sample were granted in writing by the Exploration and Development Department of the Xingzichuan Oil Production Plant (authorization dated 30 October 2022). No governmental permits were required because the work relied exclusively on existing industry-owned cores housed outside protected areas and did not involve living organisms, human participants, or endangered species.

### 3.2 Solvent extraction and fractionation

The core shale samples were washed and cut off to remove the external surfaces before crushing. The samples were then crushed to a particle size of less than 100 mesh. The crushed powder was Soxhlet-extracted using a dichloromethane and methanol solution (9:1 v:v) for 72 h to obtain the extractable organic matter (EOM). The resulting EOM was filtered to remove any impurities, de-asphalted with cold *n*-hexane, and the precipitated asphaltenes were weighed. Subsequently, the maltene fraction was separated into subfractions of saturated hydrocarbons, aromatic hydrocarbons, and resins using activated silica gel and alumina column chromatography, with sequential elution using *n*-hexane, dichloromethane:methanol (1:1 v:v), and anhydrous ethanol:dichloromethane (3:1 v:v).

### 3.3 Gas chromatography-mass spectrometry

Analyses of the saturated and aromatic hydrocarbon fractions were performed using an Agilent 6890N gas chromatograph coupled to a 5975 mass-selective detector (MSD). The GC was equipped with an HP-5MS fused-silica capillary column (60 m × 0.25 mm i.d., 0.25 μm film). The oven temperature program started at 50 °C, increased at 3 °C· min^−1^ to 310 °C, and was held for 15 min. Helium was used as the carrier gas at a constant flow rate of 1.0 mL·min^−1^. Samples were introduced via an autosampler in split mode; 1 μL of sample solution (extracted fractions diluted with n-hexane to a final analyte concentration of 10–100 μg·mL^−1^) was injected. The mass spectrometer operated under electron ionization (70 eV), with the ion source and quadrupole temperatures set at 230 °C and 150 °C, respectively. Data were acquired in full-scan mode over m/z 50–580 and, where appropriate, by selected-ion monitoring (SIM) to enhance the detection of hopanes and rearranged hopanes.

For quantification, each sample was spiked with D_50_-*n*-tetracosane (*n*-C_24_D_50_) to determine *n*-alkane concentrations and with 5α-androstane for sterane and hopane biomarkers; the final concentrations of the internal standards in the injection solution were 0.4701 μg·μL^−1^ and 0.1049 μg·μL^−1^, respectively. Concentrations and molecular parameters were calculated from the peak-area ratios relative to the corresponding internal standard.

### 3.4 Statistical analysis

All statistical analyses were performed using Microsoft Excel 365 (Microsoft Corporation, Redmond, WA, USA) with the Real Statistics Resource Pack add-in (version 9.5.5). To avoid the statistical closure problem associated with compositional data, a ternary diagram was used to visualize the relative proportions of C₃₀ hopanes and a C₃₀ Rearranged Hopane Index (RHI) for quantitative analysis.

Spearman’s rank correlation analysis (r_s_), a non-parametric method that is robust to small sample sizes and non-normally distributed data, was employed to assess monotonic relationships between key biomarker parameters. This analysis was applied to investigate the relationships between various rearranged hopane ratios (e.g., C₃₀D/C₃₀H, C₃₀E/C₃₀H) and between the RHI and multiple thermal maturity proxies, including the calculated vitrinite reflectance (Rc; derived from MPI-1), Ts/Tm, and C₂₉ sterane isomerization ratios. For maturity trend analysis, the statistical outlier sample X762 was excluded to better evaluate the underlying trend of the main sample population.

The two-tailed significance level was set at α = 0.05. Correlation strength was interpreted using conventional thresholds for the absolute value of the Spearman’s coefficient (|r_s_|): strong (≥0.7), moderate (0.5–0.7), or weak (<0.5). Linear trend lines and coefficients of determination (R2) were generated in the plots for visual purposes only, to illustrate the general trends. All detailed statistical results, including correlation coefficients and p-values, are provided in the Supplementary Information (S2 Table).

## 4 Results and discussion

### 4.1 *n*-Alkane and acyclic isoprenoid characteristics

**Fig 2:**
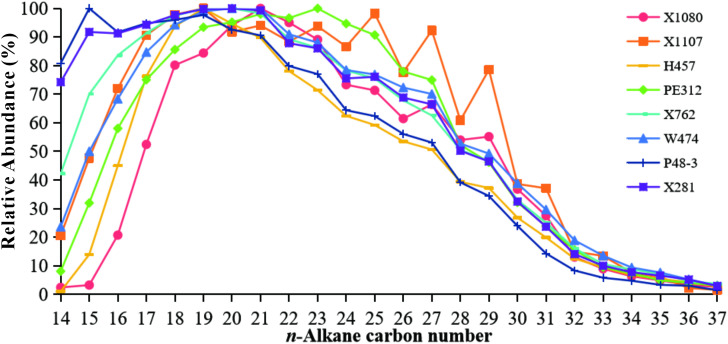
Relative distribution of *n*-alkanes (based on *m*/*z* 85 data) in the Chang 9 shales.

The results revealed the presence of *n*-alkanes ranging from *n*-C_14_ to *n*-C_37_ in all the shale samples from the Chang 9 member (Fig 2). The *n*-alkanes displayed a smooth unimodal distribution of C_14–37_ with maxima centered around *n*-C_19_ to *n*-C_21_ [31,32]. This distribution pattern is indicative of a relatively mature stage [33]. Sample PE312 exhibited a slightly elevated maxima at *n*-C_23_. The odd–even preference (OEP) and the carbon preference index (CPI) were close to 1 for the majority of the samples (Table 1), indicating a minimal OEP for *n*-alkane. However, sample X1107 exhibited notably elevated OEP (1.41) and CPI (1.5) values (Table 1), indicative of an odd-over-even *n*-alkane preference for *n*-alkanes spanning *n*-C_19_ to *n*-C_30_ (Fig 2). This result indicates that sample X1107 was relatively less mature. Notably, samples X1080 and X1107 exhibited medium-heavy molecular weight ratios of *n*-alkanes, as indicated by the ratio of ((*n*-C_21_ + *n*-C_22_)/(*n*-C_28_ + *n*-C_29_)) and ∑*n*- C_21_^-^/∑*n*-C_22_^+^ (0.67–0.76 and 0.98–1.38) compared to others (0.82–2.0 and 1.7–2.6) (Table 1). Furthermore, the terrigenous to aquatic *n*-alkane ratios [34] for samples X1080 and X1107 were 1.06 and 0.87, respectively, considerably higher than the average ratio of 0.54 observed for the remaining samples (Table 1). It can thus be posited that samples X1080 and X1107 likely contained a greater proportion of land-derived organic matter during the process of sedimentation than the remaining samples, which exhibited a combination of aquatic plants and higher organism contributions.

**Fig 3:**
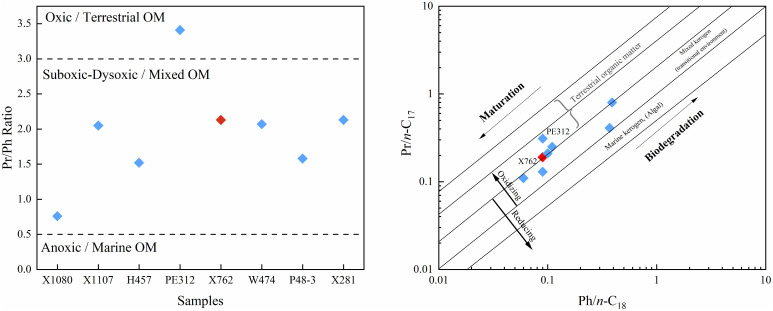
Acyclic isoprenoid parameters illustrating the organic matter source and redox conditions of the Chang 9 source rocks. **(a)** Pr/Ph ratios, showing that most samples were deposited under suboxic-to-anoxic conditions, with the notable exception of sample PE312 suggesting an oxic or terrestrial-influenced setting. **(b)** Cross-plot of Pr/*n*-C_17_ versus Ph/*n*-C_18_, showing a transitional trend where samples plot within the ‘Mixed organic sources’ field and extend into the ‘Terrestrial organic matter’ field. No samples fall within the ‘Marine organic matter’ field. Crucially, sample PE312, which appeared anomalous in Fig 3a, now plots unequivocally within the ‘Terrestrial organic matter’ field. This provides definitive evidence that its high Pr/Ph ratio is primarily controlled by the dominant input of terrestrial organic matter rather than reflecting a truly oxic depositional environment. Furthermore, the data points spread across the oxidizing-to-reducing trend lines, indicating that this organic matter was deposited under fluctuating, oxygen-limited conditions. Interpretive bands modified after Shanmugam (1985) [38] and Peters et al. (2005) [33]. Sample PE312 is explicitly labeled for clarity, and sample X762 (red symbol) is highlighted.

Pristane (Pr) and phytane (Ph) are important acyclic isoprenoids, and their ratios, such as Pr/Ph, Pr/*n*-C17, and Ph/*n*-C18, are widely used as indicators of redox conditions, organic matter input, and thermal maturity [31,35–38]. The Pr/Ph ratios of the Chang 9 samples ranged from 0.76 to 3.4 (Table 1). As shown in Fig 3a, most samples fell within the suboxic–dysoxic range (Pr/Ph = 0.5–3.0), while one sample (PE312) showed plots in the oxic field (Pr/Ph > 3.0), suggesting variable degrees of terrestrial influence among samples.

However, a more precise interpretation is provided by the Pr/*n*-C_17_ versus Ph/*n*-C_18_ cross-plot (Fig 3b), a diagnostic tool widely used for its ability to effectively deconvolve the interconnected effects of organic matter source and redox conditions [38]. The plot shows a clear transitional trend for the Chang 9 samples, which originate from a ‘Mixed organic sources’ setting, characterized by a significant aquatic contribution (as evidenced by n-alkane maxima at *n*-C_19_–C_21_ and TAR ≤ 1; Table 1), and extend clearly into the ‘Terrestrial organic matter’ field. Importantly, this source trend corresponds well with a redox gradient, where samples in the ‘Mixed’ field represent suboxic-dysoxic conditions, while the progression towards the ‘Terrestrial’ field aligns with increasingly oxic conditions. This framework helps to explain the characteristics of the “anomalous” sample PE312, which appeared as a strongly oxic outlier in Fig 3a. In Fig 3b, this ambiguity is clearly resolved, as PE312 is located at the end of this trend, plotting clearly within the ‘Terrestrial organic matter’ field and in the ‘Oxidizing’ position. This suggests that its high Pr/Ph ratio can be primarily attributed to the combined effects of both a dominant terrestrial organic matter input and a more oxic depositional environment. While other factors such as thermal maturity or biodegradation can influence these parameters (as indicated in Fig 3b), the consistent alignment of the data along the source-redox trend indicates that this interplay is the principal control for the Chang 9 sample suite. Consequently, the apparent contradictions in the dataset are best explained by a source-redox system, where increased terrestrial input correlates with more oxic conditions.

### 4.2 Sterane and diasterane characteristics

**Fig 4:**
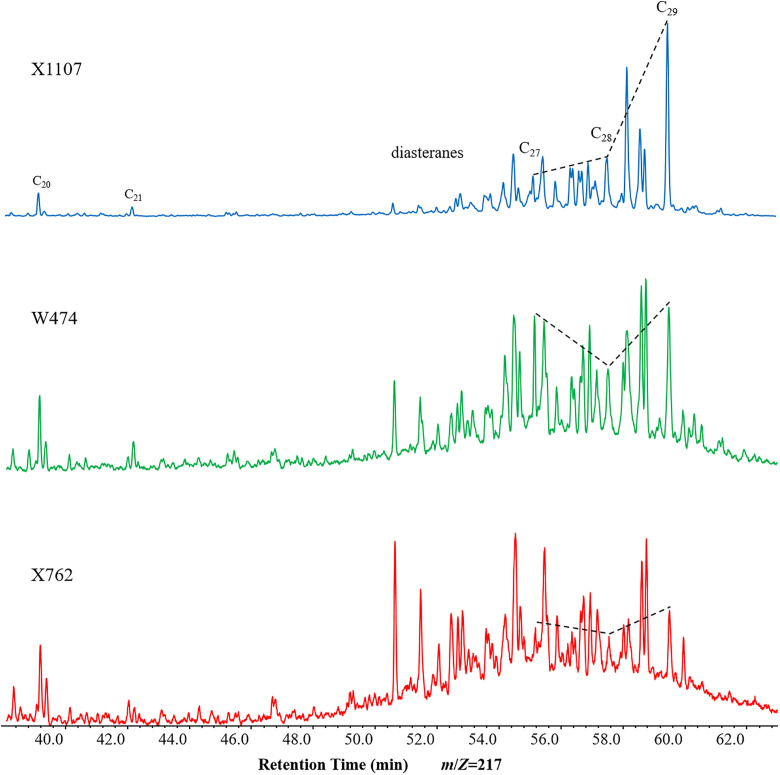
Partial *m*/*z* 217 mass chromatograms showing the distribution of steranes and diasteranes in typical samples.

**Fig 5:**
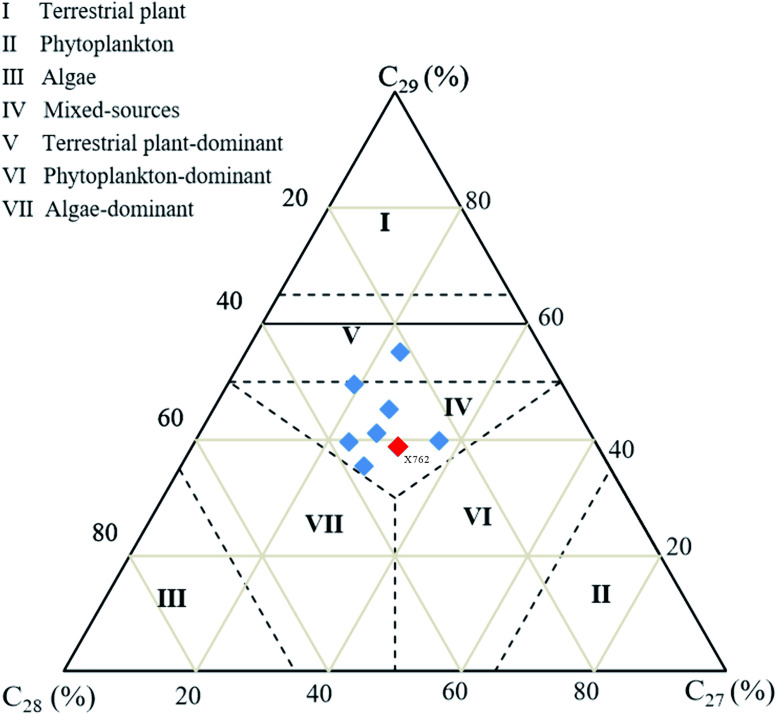
C_27_-C_28_-C_29_ regular sterane ternary diagram for the Chang 9 source rocks, used to infer organic matter input. The anomalous sample X762 (red symbol) is highlighted to distinguish it from the main group (blue symbols); note its position within the main data cluster.

Steranes are widely distributed in source rocks and crude oils. The relative contents of C_27_, C_28_, and C_29_ steranes from different sources are commonly used to determine the nature of the parent organic materials [20,31,39]. The *m/z* 217 chromatographic analysis of representative Chang 9 source rock samples revealed differences in distribution patterns and relative abundances (Fig 4, Table 2). The proportions of C_27_, C_28_, and C_29_ regular steranes are presented in the ternary diagram in Fig 5. In general, the ααα 20R C_27_–C_28_–C_29_ distribution exhibited a slight preference for C_27_ > C_28_ < C_29_, resulting in a “V” shaped profile in the *m*/*z* 217 mass chromatograms (Fig 4). Sample X1107 exhibited a distinctive inverted “L” type distribution, accompanied by an elevated C_29_ contribution of 55.1%, situated in the upper region of the ternary diagram.

**Table 2:** Steranes and terpenes parameters of the Chang 9 shales.

Samples	C_27_%	C_28_%	C_29_%	Dia/St	C_29_ ββ/(αα + ββ)	C_29_ 20S/(20S+20R)	C_27-29_ ββ/αα	C_30_D/C_30_H	C_30_E/C_30_H	C_29_D/C_30_H	C_29_Ts/C_30_H	Ga/C_30_H	Ts/Tm	MPI1	Rc	A	B	C	RHI
X1080	23.4	36.7	39.8	0.16	0.46	0.44	0.86	0.07	0.04	0.05	0.16	0.20	0.76	0.74	0.84	0.90	0.04	0.06	0.10
X1107	21.7	23.2	55.1	0.15	0.43	0.31	0.63	0.06	0.02	0.03	0.07	0.10	0.11	0.54	0.73	0.93	0.02	0.06	0.08
H457	30.2	31.0	38.8	0.29	0.49	0.47	1.05	0.50	0.17	0.25	0.36	0.13	2.8	0.75	0.85	0.60	0.10	0.30	0.40
PE312	31.4	19.1	49.6	0.30	0.49	0.51	1.29	0.81	0.28	0.36	0.44	0.17	4.4	0.73	0.84	0.48	0.13	0.39	0.52
X762	36.9	27.6	35.5	0.51	0.47	0.59	1.45	4.08	1.46	1.42	0.72	0.45	6.7	0.85	0.91	0.15	0.22	0.62	0.84
W474	37.1	23.2	39.7	0.26	0.51	0.46	0.94	0.28	0.09	0.15	0.28	0.09	2.1	0.66	0.80	0.73	0.06	0.20	0.26
P48-3	28.3	26.5	45.3	0.11	0.55	0.51	1.25	0.49	0.11	0.32	0.37	0.15	3.6	0.68	0.81	0.62	0.07	0.31	0.38
X281	32.2	26.7	41.2	0.31	0.58	0.51	1.32	1.14	0.34	0.42	0.63	0.27	6.5	0.76	0.86	0.40	0.14	0.46	0.60

Dia/St: Diasterane/sterane; C_30_D: C_30_ 17α(H)-diahopane; C_30_H: C_30_ 17α(H)-hopane; C_30_E: C_30_ early eluting rearranged triterpane; C_29_D: C_29_ 17α(H)-diahopane; C_29_Ts: C_29_ 18α-30-norneohopane; Ga: Gammacerane; Ts: 18α-trisnorneohopane; Tm: 17α-trisnorhopane; MPI1: (1.5 × (3-MP + 2-MP)/(9-MP + 1-MP + P)); Rc = [(0.6 × MPI1) + 0.4]; A: C_30_H/(C_30_E+C_30_D + C_30_H); B: C_30_E/(C_30_E+C_30_D + C_30_H); C: C_30_D/(C_30_E+C_30_D + C_30_H); RHI= (C_30_E + C_30_D)/(C_30_E+C_30_D + C_30_H).

This distribution infers a greater contribution from terrigenous plants (Figs 4–5, Table 2). The C_29_ steranes exhibited a slightly higher relative abundance (38.8–55.1%) in the majority of samples, followed by C_27_ (21.7–37.1%) and C_28_ (19.1–36.7%). This indicates mixed organism input, and a slightly higher abundance of C_27_ steranes (36.9%) than that of C_29_ steranes (35.5%) was observed in sample X762 (Fig 5, Table 2). The ratio of diasteranes to regular steranes is generally consistent across samples; however, their relative abundances vary. Diasterane abundance in the samples was relatively low, with a diasterane/sterane value of 0.15. Nevertheless, diasteranes constitute significant components in some samples, with the highest diasterane/sterane ratio of 0.51 observed in sample X762 (Fig 4, Table 2).

**Fig 6:**
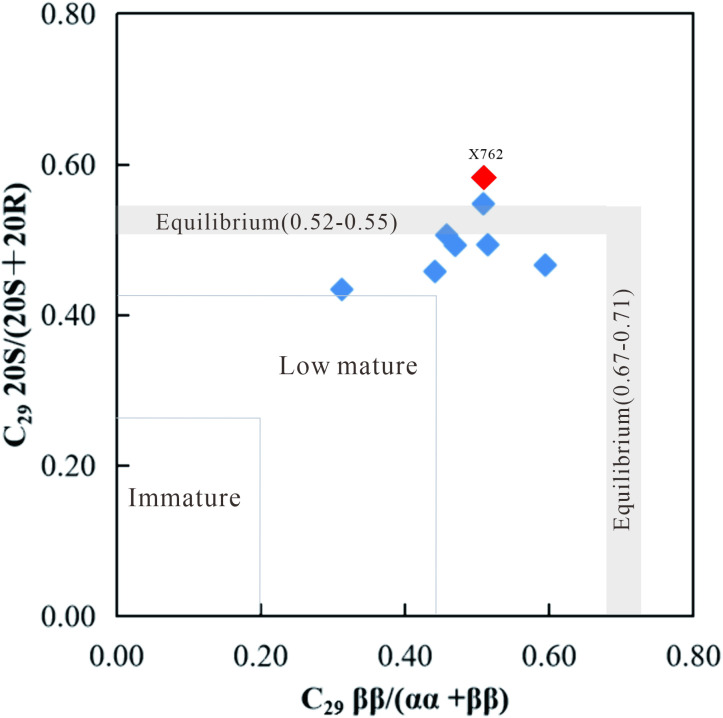
Cross-plot of sterane thermal maturity parameters C_29_ 20S/(20S + 20R) vs. C_29_ ββ/(αα + ββ) for the Chang 9 source rocks. The anomalous sample X762 (red symbol) is highlighted to distinguish it from the main group (blue symbols), plotting within the mature end of the sample distribution.

The sterane maturity parameters, C_29_ 20S/(20S + 20R) and C_29_ ββ/(αα + ββ), exhibited ranges of 0.43–0.58 and 0.31–0.59, respectively, in the studied samples (Table 2). The ratio values were close to or at their thermal equilibrium values (Fig 6), indicating that the shale samples of the Chang 9 member had reached the thermal maturity of the peak oil window [39].

### 4.3 Hopane characteristics

#### 4.3.1 Occurrence of rearranged hopanes.

**Fig 7:**
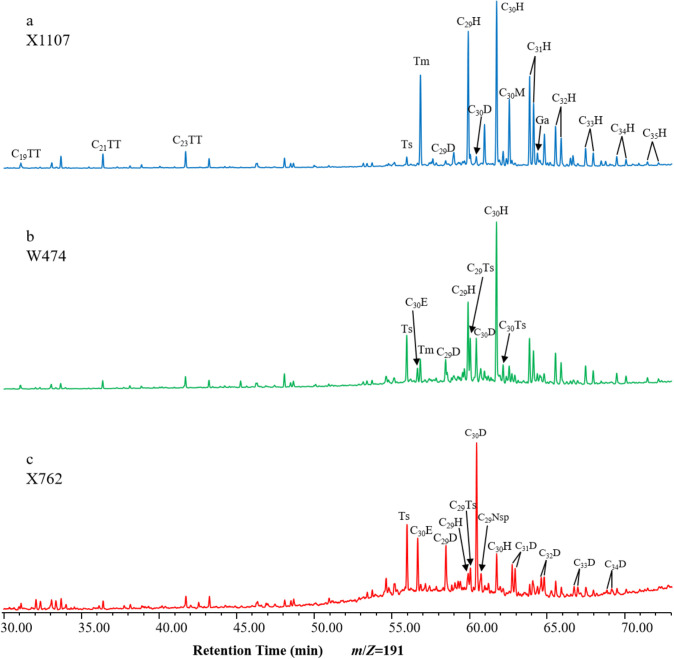
Partial m/z 191 mass chromatograms showing the distribution of tricyclic terpanes, hopanes, diahopanes and rearranged hopanes in typical samples.

In *m*/*z* 191 mass chromatograms of saturated hydrocarbons derived from shale samples of the Chang 9 source rocks in the Ansai area, both regular and rearranged hopanes were identified, exhibiting notable variations in distribution between samples. Four series of rearranged hopanes were identified, including the 17α(H)-diahopanes (D series), 18α(H)-neohopanes (Ts series), early-eluting rearranged hopanes (E series), and relatively small amounts of 28-nor-spergulanes (Nsp series) (Fig 7) [40]. These biomarkers were identified through comparison of their relative retention times and mass spectra with those reported in the literature [10]. In general, the most abundant hopanes are typically the D-series, which have a similar distribution to regular hopanes but consistently elute earlier at the same carbon number. D-series biomarkers were present in the carbon number range C_27_–C_35_, except C_28_. Paired peaks of C_31_–C_35_D, representing both S and R isomers, were observed in the shale samples of the Chang 9 source rock (Fig 7c).

However, only C_29_D and C_30_D were consistently detected in all samples. The Chang 9 source rock extracts showed lower abundance of 18α(H)-neohopanes than 17α(H)-diahopanes. The former were primarily composed of C_27_, C_29_ and C_30_ homologues (Ts, C_29_Ts, and C_30_Ts, respectively). The early-eluting rearranged hopanes eluted two carbon numbers earlier than regular hopanes as a consequence of their shorter and more compact molecular structures. However, the E-series had low relative abundances and absolute concentrations, and only the C_30_E homolog was clearly detected. Its peak position was between Ts and Tm in the *m*/*z* 191 mass chromatograms and much closer to Tm. The Nsp series, with the exception of C_29_Nsp, were rarely detected in this study and were relatively low in abundance.

Nevertheless, the occurrence of all four hopane rearrangements in a single geological sample is extremely rare. Furthermore, it is also unusual for the abundance of C_30_E to exceed that of its neighbors, including Ts and Tm, in the *m*/*z* 191 mass chromatograms [32]. Sample X762 displayed the most anomalous composition and distribution of pentacyclic triterpenes, with very few normal 17α(H)-hopane series and a prevalence of rearranged hopanes. Notably, all four series were detected (Fig 7).

#### 4.3.2 Distribution of rearranged hopanes.

The mass chromatograms for *m*/*z* 191 demonstrated a notable variation in the distribution of rearranged hopanes among the samples within the Chang 9 member. Based on this observation, the Chang 9 source rocks were classified into three main types. The first type, exemplified by X1107, was characterized by a predominance of C_30_ hopanes (C_30_H) and a normal distribution pattern of C_31_–C_35_ homohopane compounds, which decreased in abundance with increasing carbon number. This sample type exhibited a lower relative abundance of Ts than Tm. The rearranged hopanes were primarily composed of low-abundance C_29_D, C_29_Ts, C_30_D, and C_30_M (Fig 7a). The ratios of C_30_D/C_30_H and Ts/Tm were relatively low, with values of 0.06–0.07 and 0.11–0.76, respectively (Table 2). In contrast, sample X762 was characterized by strong dominance of C_30_D and a relatively complete D series. The ratio of C_30_D to C_30_H was as high as 4.1 (Fig 7c, Table 2). Furthermore, the samples contained a substantial amount of Ts, C_29_D, C_30_E, and diahopanes (C_31_–C_34_), which exhibited markedly higher abundances than the corresponding 17α(H)-homohopanes (Fig 7c). In particular, the C_30_E isomer was more abundant than C_30_H in sample X762, with a C_30_E/C_30_H ratio of 1.46 (Table 2). Notably, this sample also exhibited elevated abundances of diasteranes (Fig 4, Table 2). To avoid the leverage effect, X762 was plotted as a separate group (in red) in all the figures. The third source rock type, exemplified by W474, demonstrated a pronounced increase in the diversity and relative abundance of rearranged hopane compounds detected, compared to the first sample type. Additionally, Ts series and C_30_E were found to have high relative abundances (Fig 7b).

**Fig 8:**
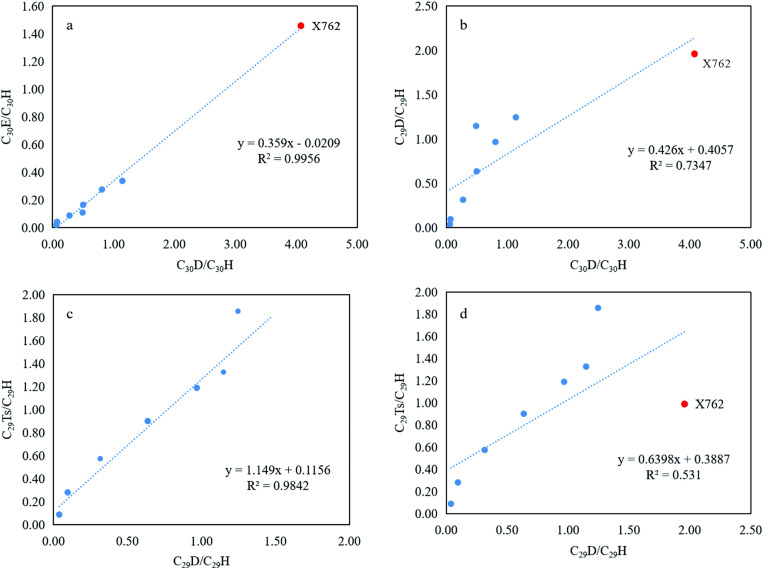
Correlation plots for key rearranged hopane parameters in the Chang 9 source rocks. **(a)** C_30_D/C_30_H vs. C_30_E/C_30_H. **(b)** C_29_D/C_29_H vs. C_30_E/C_30_H. **(c)** C_29_Ts/C_29_H vs. C_29_D/C_29_H, with the linear regression excluding X762. **(d)** The same plot as **(c)**, but with the regression including X762 to illustrate its strong leverage effect. The anomalous sample X762 (red symbol) is highlighted to distinguish it from the main group (blue symbols).

The distribution relationships of the rearranged hopanes from the Chang 9 source rocks were further analyzed. The associated hopane parameters were calculated and are presented in Table 2 for purposes of analysis. As illustrated in Fig 8a, the ratio of C_30_E/C_30_H exhibited a significant positive monotonic correlation with that of C_30_D/C_30_H (Spearman’s r_s_ = 1.0000, p < 0.0001), with a linear trend line showing a high coefficient of determination (R^2^ = 0.9956) and an intercept of less than 0.0209, indicating similar biological origins and formation conditions for these two series. Sample X762 exhibited considerably higher ratios than other samples across multiple hopane parameters; for example, the exclusion of X762 from the analysis results in a strong monotonic correlation between C_29_D/C_29_H versus C_30_D/C_30_H (Spearman’s r_s_ = 0.9286, p = 8.63 × 10^−4^) with a moderate linear fit (R^2^ = 0.735) (Fig 8b) and a slightly higher value was observed in sample X281. Furthermore, the plots of C_29_Ts/C_29_H and C_29_D/C_29_H demonstrated a robust monotonic correlation (Spearman’s r_s_ = 0.9643, p = 0.0001), supported by a strong linear fit (R^2^ = 0.984) (Fig 8c). However, including X762 significantly weakened the linear fit (R^2^ = 0.531, Spearman’s r_s_ = 0.8571, p = 0.0065) (Fig 8d), prompting a detailed examination of the geochemical drivers behind this anomaly.

Sample X762 exhibited exceptionally high rearranged-hopane ratios relative to the rest of the Chang 9 dataset, with C_30_D/C_30_H = 4.08, C_30_E/C_30_H = 1.46, and C_29_D/C_30_H = 0.42 (Table 2). We attributed this anomaly primarily to enhanced mineral acid-catalyzed rearrangement during early diagenesis in a clay-rich lacustrine microenvironment, with a secondary contribution from differences in organic-matter input and water-column stratification. This interpretation was supported by three lines of evidence. (1) Clay-catalysis proxies were markedly elevated in X762: the diasterane/sterane ratio was the highest of the set (Dia/St = 0.51), Ts/Tm was extremely high (6.7, comparable to X281 = 6.5), and C_29_Ts/C_30_H was also high (0.42). These features were classically associated with acid-catalyzed rearrangement on clay mineral surfaces [31,33]. (2) The thermal maturity was not a dominant control. Although X762 showed relatively high maturity based on several proxies—including MPI1 (0.85), C₂₉ 20S/(20S+20R) sterane isomerization (0.59), and its calculated vitrinite reflectance (Rc) of approximately 0.91 (derived from MPI-1 using the equation Rc = 0.6 × MPI-1 + 0.4 [41])—this alone cannot account for the anomaly. Crucially, a sample with similar or even greater maturity signatures (X281: C_29_ 20S/(20S+20R) = 0.62; Ts/Tm = 6.5) displayed a much lower C₃₀D/C₃₀H ratio of 1.14. This sharp contrast indicates that maturity was not the primary driver of the extraordinary enrichment of rearranged hopanes observed in sample X762. (3) Facies and input indicators point to a stratified lacustrine setting with a comparatively stronger algal/bacterial contribution, which provides hopanoid precursors and favors preservation: X762 had the highest gammacerane content relative to C_30_ hopane (Ga/C_30_H = 0.72), the lowest or near-lowest Pr/*n*-C_17_ (0.19) and Ph/*n*-C18 (0.09), and a relatively low TAR (0.51). Taken together, these observations favor mineral-catalyzed rearrangement as the dominant driver of the anomaly, amplified by facies and organic-matter input effects.

### 4.4 Primary influencing factors of rearranged hopanes

#### 4.4.1 Organic matter/acid catalysis.

**Fig 9:**
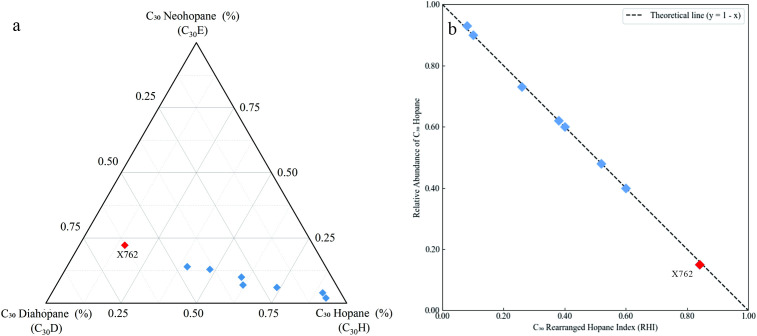
Compositional representation of the C_30_ triterpane system for the Chang 9 source rocks. **(a)** Ternary plot showing the fractional contributions of C_30_ hopane (C_30_H), C_30_ diahopane (C_30_D), and C_30_ neohopane (C_30_E), normalized to C_30_H + C_30_D + C_30_E = 1. **(b)** Relationship between the C_30_ Rearranged Hopane Index (RHI = (C_30_D + C_30_E)/(C_30_D + C_30_E + C_30_H)) and the relative abundance of C_30_ hopane (C_30_H/(C_30_D + C_30_E + C_30_H)); the dashed line represents the theoretical mass-balance complement (y = 1 – **x)**. Values are derived from m/z 191 mass chromatograms. The anomalous sample X762 (red symbol) is highlighted to distinguish it from the main group (blue symbols).

The conventional view holds that rearranged hopanes share identical biological precursors with regular hopanes, originating primarily from bacteriohopanetetrol [10,42]. This implies a precursor–product relationship in which regular hopanes are converted into rearranged hopanes during diagenesis [10,43]. To investigate this relationship in the Chang 9 samples while avoiding the statistical pitfalls of compositional data closure, we visualized the relative proportions of C₃₀ diahopane (C₃₀D), C₃₀ neohopane (C₃₀E), and regular C₃₀ hopane (C₃₀H) using a ternary diagram (Fig 9a).

The data points form a clear linear trend extending from the C₃₀H apex towards the C₃₀D–C₃₀E baseline. This distribution strongly supports a systematic conversion process in which the abundance of C₃₀H decreases as those of C₃₀D and C₃₀E increase. To quantify the extent of this conversion, we defined a C₃₀ Rearranged Hopane Index (RHI) as (C₃₀D + C₃₀E)/ (C₃₀D + C₃₀E + C₃₀H). As shown in Fig 9b, there was a mathematically defined inverse relationship between the RHI and the relative proportion of regular C₃₀ hopane (%C₃₀H). This robust relationship confirms that the formation of rearranged hopanes in the Chang 9 source rocks occurred at the expense of regular hopanes. This establishes the crucial premise that this transformation is driven by geological factors, such as clay-mediated acid catalysis.

**Fig 10:**
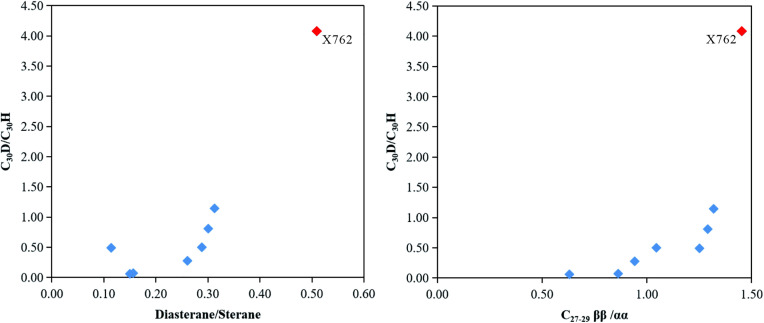
Crossplots of rearranged hopanes and sterane ratios: (a) C_30_D/C_30_H versus Diasterane/Sterane; (b) C_30_D/C_30_H versus C_27-29 _ββ/αα. The anomalous sample X762 (red symbol) is highlighted to distinguish it from the main group (blue symbols).

High diasterane/sterane ratios are typical of petroleum generated from clay-rich source rocks, and the diasterane/sterane ratio shows a good relationship with the diahopane/hopane or early-eluting series/hopane ratio [3,31]. Similarly, it was found that the samples with high abundances of rearranged hopanes also had high abundances of diasteranes (Figs 4 and 7, Table 2). The same feature was reported by Ruble TE [42]. Furthermore, samples with high abundances of regular sterane isomers with ββ configuration also had relatively high abundances of rearranged hopanes (Figs 4 and 7). As shown in Fig 10, the C_30_D/C_30_H ratios increased with increasing Dia/St and C_27-29_ ββ/αα ratios. Previous studies have reported that sterane isomerization is controlled not only by thermal maturity, but probably also by the catalysis of active clay (e.g., kaolinite), mineral matrix (e.g., gypsum), and hypersaline environments [44–46].

Previous reports suggest that the high abundance of 17α(H)-diahopanes in oils from the Korea Bay Basin may be closely related to the input of terrigenous land plant material [14] and/or may be generated from bacterially reworked terrigenous organic matter [2,32]. However, most of the Chang 9 samples have mixed sources, and the contribution of terrigenous land plant material appears to be insignificant. Sample X1107 had the lowest abundance of rearranged hopanes, despite having the highest input of terrigenous organic matter (Table 2). This suggests a limited role of terrigenous organic matter in the formation of rearranged hopanes in the Ansai area.

#### 4.4.2 Sedimentary environment.

**Fig 11:**
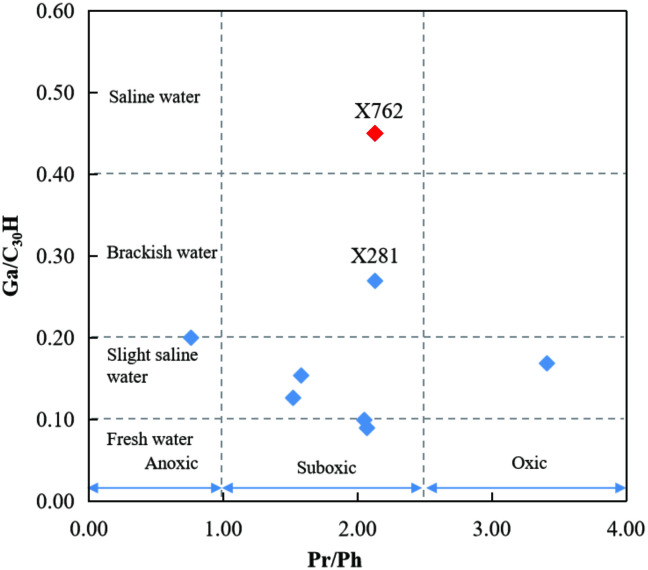
Cross-plot of Ga/C_30_H vs. Pr/Ph used to discriminate source rock depositional environments. The anomalous sample X762 (red symbol) is highlighted to distinguish it from the main group (blue symbols).

Sedimentary environments can contain different assemblages of organisms, resulting in different biomarkers in sediments [31]. The redox conditions and salinity of sedimentary and diagenetic waters influence the relative abundance of C_30_D [10,14,31]. According to Moldowan et al. [14], the four rearranged hopane series are formed by clay-mediated acid catalysis of bacteriohopanoid precursors under oxic or suboxic depositional conditions. Xiao et al. [17] concluded that high-salinity water conditions would inhibit the proliferation of rearranged hopane precursors. The environments reflected by Ga/C_30_H and Pr/Ph varied greatly in this study. The Chang 9 member was mainly deposited in fresh to slightly saline water and suboxic sedimentary environments. In contrast, samples from X762 and X281 were formed in brackish to saline water environments (Fig 11).

**Fig 12:**
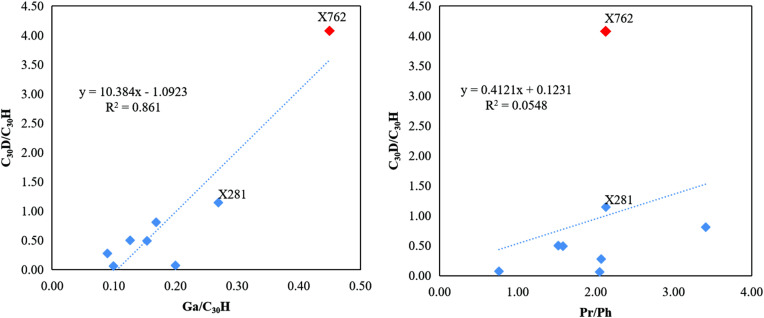
Relationship between the C_30_D/C_30_H ratio and depositional environment parameters. **(a)** C_30_D/C_30_H vs. Ga/C_30_H. **(b)** C_30_D/C_30_H vs. Pr/Ph. The anomalous sample X762 (red symbol) is highlighted to distinguish it from the main group (blue symbols).

Zhang et al. [47] reported a positive relationship between C_30_D/C_30_H and Pr/Ph in Yanchang source rocks from the Wuqi–Zhidan area. They suggested that a high to very high abundance of C_30_D indicates a shallow-oxic environment, while a low abundance of C_30_D may indicate an anoxic sedimentary environment. The C_30_D/C_30_H ratios in the Upper Triassic Chang 9 member show good correlation with the gammacerane index but poor correlation with Pr/Ph (Fig 12, Table 2). Samples X762 and X281, which were deposited in saline to brackish and suboxic sedimentary environments, respectively, showed relatively high abundances of rearranged hopanes. This suggests a positive correlation between the water salinity of the depositional environment and the high content of rearranged hopanes. Other studies [42,48] have also found that a saline water environment promotes the formation of rearranged hopanes. However, the redox state of the sedimentary environment appears to have had little effect on the rearranged hopanes in the Chang 9 member. Both oxic and anoxic sedimentary environments will inhibit the enrichment of rearranged hopanes. For example, sample PE312 was deposited under more oxic conditions than X762 and X281 (Fig 11), but contained fewer rearranged hopanes relative to hopanes. The Ga/C_30_H and C_30_D/C_30_H values for sample X762 showed a substantially different range than that of the other samples. It is suggested that the rearranged hopanes in sample X762 are predominantly derived from a specific sedimentary environment in which the presence of organisms rich in precursor molecules favored their synthesis. A strong positive trend between C_30_D/C_30_H and Ga/C_30_H (Spearman’s r_s_ = 0.6904, p = 0.0579, R^2^ = 0.861) indicates that hopane rearrangement was influenced by water salinity, and the lack of correlation between C_30_D/C_30_H and Pr/Ph (Spearman’s r_s_ = 0.6369, p = 0.0895, R^2^ = 0.0548) means that redox conditions did not determine diahopane content (Fig 12).

#### 4.4.3 Thermal maturity of organic matter.

Thermal maturity is a critical factor influencing the rearrangement of hopanes and steranes during diagenesis. In our samples, a positive correlation was observed between the abundances of diasteranes and rearranged hopanes (Figs 4 and 7, Table 2), suggesting similarities in their formation controls. Generally, increasing thermal maturity is thought to favor isomerization and rearrangement reactions, as the rearranged products are often thermodynamically more stable [3,46]. Molecular mechanics calculations support this, predicting a stability order of 17α(H)-diahopanes > 18α(H)-neohopanes > 17α(H)-hopanes [10,18].

**Fig 13:**
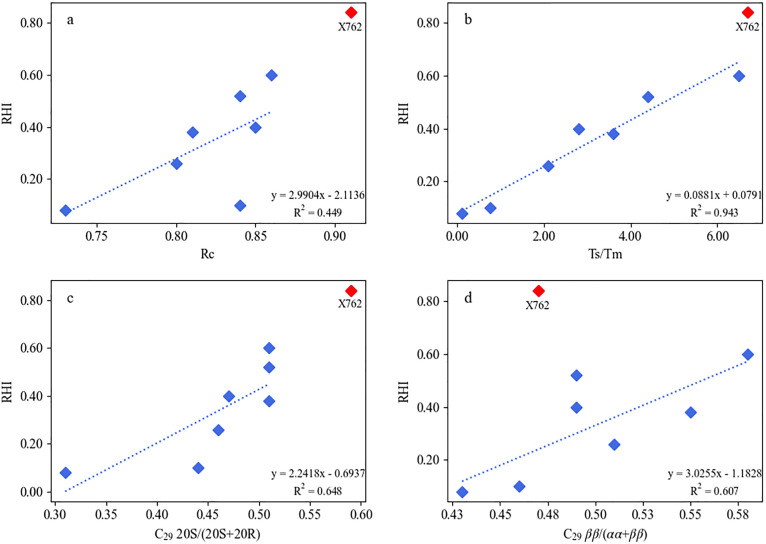
Relationships between rearranged hopane abundance (RHI) and multiple thermal maturity proxies for the Chang 9 shales. **(a)** RHI versus Rc (Ro-equivalent calculated from MPI-1; **(b)** RHI versus Ts/Tm; **(c)** RHI versus C_29_ sterane 20S/(20S+20R); **(d)** RHI versus C_29_ sterane ββ/(αα + ββ). RHI = (C_30_D + C_30_E)/(C_30_D + C_30_E + C_30_H). Sample X762 is highlighted as a red diamond. Dashed lines show linear fits computed after excluding X762; R^2^ values refer to these fits. Note that Ts belongs to the rearranged hopane family and is included here for comparison only.

To robustly evaluate the impact of maturity, and in response to the valid concern that using single maturity proxies or burial depth across different wells can be misleading, we conducted a multi-proxy analysis. We assessed the relationship between the C₃₀ RHI and four different maturity parameters derived from aromatic, triterpane, and sterane compounds (Fig 13). The outlier sample X762, which exhibited the highest RHI, was excluded from the correlation analysis to assess the general trend of the majority of the samples.

The results revealed a clear and consistent positive trend between the RHI and all four maturity proxies. Spearman’s rank correlation analysis confirmed that these relationships were statistically significant. The strongest correlation was observed between RHI and the Ts/Tm ratio (Fig 13b; Spearman’s r_s_ = 0.964, p < 0.001), followed by the C₂₉ 20S/(20S+20R) sterane isomerization ratio (Fig 13c; Spearman’s r_s_ = 0.893, p = 0.003). Significant positive correlations were also found with the C₂₉ ββ/(ββ + αα) sterane ratio (Fig 13d; Spearman’s r_s_ = 0.714, p = 0.047) and the calculated vitrinite reflectance, Rc (Fig 13a; Spearman’s r_s_ = 0.714, p = 0.047). This consistent trend across multiple, independent maturity indicators provides strong evidence that, for the main population of Chang 9 shales studied, increasing thermal maturity is a primary factor driving the formation of rearranged hopanes.

However, the non-linear nature of this control becomes evident when considering the full dataset. While the RHI generally increased with maturity, the trend was not uniform, and samples with similar high maturity levels can exhibit vastly different RHI values. For example, sample X281 and the outlier X762 had comparable high maturities (e.g., Rc ≈ 0.86–0.91), but their RHI values differed significantly (0.60 vs. 0.84, respectively, Table 2). This divergence at higher maturities suggests that once a certain thermal threshold is reached, the influence of thermal maturity may become secondary to other geological factors. At that stage, variations in the catalytic activity of the mineral matrix (i.e., clay content and type) or specific characteristics of the depositional environment likely become the dominant controls on the final abundance of rearranged hopanes, leading to the observed scatter in the data.

## 5 Conclusion

The Chang 9 shale samples from the Ansai area of the Ordos Basin were deposited under a spectrum of redox conditions (representing a transition from suboxic to oxic states), with a mixed organic matter input characterized by an initial significant aquatic contribution that shows a progressively increasing terrestrial influence. Comprehensive analysis identified all four major series of rearranged hopanes. Based on their distinct distribution patterns, the Chang 9 source rocks can be classified into three types: a low-maturity type dominated by regular hopanes (e.g., X1107), a highly rearranged type with an exceptionally high abundance of C₃₀ diahopane (e.g., X762), and an intermediate type. A robust precursor–product relationship was confirmed through ternary diagram analysis, which demonstrated that rearranged hopanes (C₃₀D and C₃₀E) were diagenetic products formed at the expense of regular C₃₀ hopanes (C₃₀H). The enrichment of these compounds was controlled by a complex interplay of multiple factors. The depositional conditions were critical, with higher abundances observed in more saline and suboxic environments. Thermal maturity acted as a primary driver in the early oil window, as evidenced by the significant positive correlations with multiple, independent maturity proxies. However, its influence diminished at higher maturity levels, where the catalytic properties of the mineral matrix likely became the dominant control. The comprehensive analysis of the biomarker characteristics in this study provides robust insights into the geochemical features of the Chang 9 shale. However, we acknowledge that this study is geographically focused. To verify the universality of these findings, further research incorporating data from other areas of the Ordos Basin is recommended.

## Supporting information

S1 Table Hopane isomeric alkane data and figures. (XLSX)

S2 Table Hopane ratio normality and correlation tests. (XLSX)

S3 File Mass Spectral Identification of C_30_E and C_30_D. (PDF)
