# Supplementary material for: Distribution and characteristics of rearranged hopanes in the black shale of the Chang 9 member, the Upper Triassic Yanchang Formation in the Ansai area, Ordos Basin, North China
Source: PLoS One. 2025 Dec 1;20(12):e0337076. doi: 10.1371/journal.pone.0337076 (PMC12668623; doi:10.1371/journal.pone.0337076)
Supplement: S3 File — (PDF) [file pone.0337076.s003.pdf]

Mass Spectral Identification of $C_{30}D$ and $C_{30}E$

To enhance the confidence in biomarker identification, representative mass spectra of $C_{30}E$ and $C_{30}D$ from analyzed samples are provided below.

These spectra were compared with published reference spectra to ensure accurate identification, particularly to differentiate them from thermally mature biomarkers such as Tm, with which co-elution may occur.

A. Mass Spectrum of $C_{30}D$

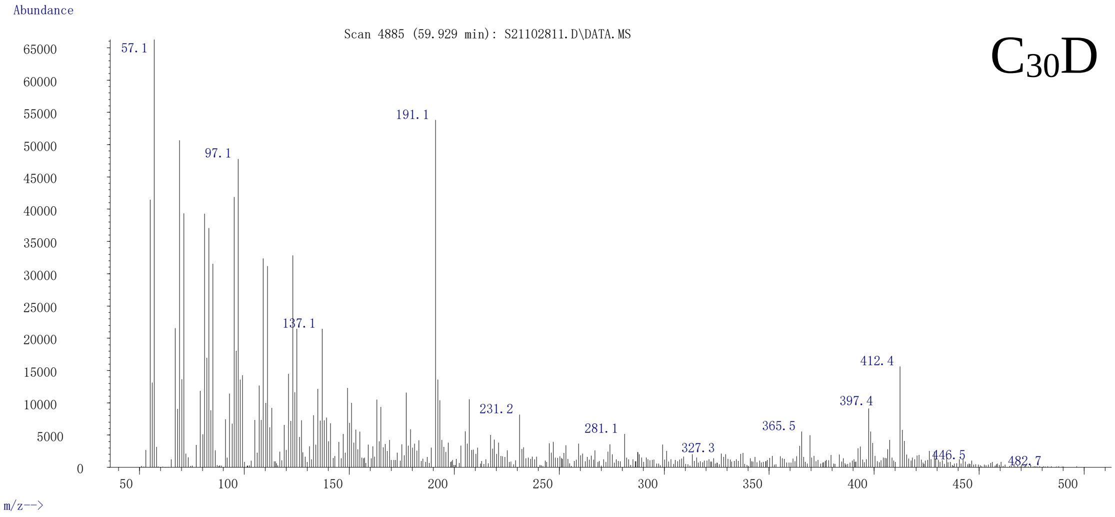

Figure 1. Representative mass spectrum of $C_{30}D$ (sample X762, retention time = 59.929 min).

The spectrum shows:

- Molecular ion at m/z 412

- Base peak at m/z 191

- Distinct diagnostic fragment ions at m/z 231 and 397

These are typical for 17alpha-diahopane ($C_{30}D$), and clearly distinguishable from $C_{30}E$ or Tm, consistent with reference spectra in Xiao et al. (2019) and Farrimond & Telnæs (1996).

B. Mass Spectrum of $C_{30}E$

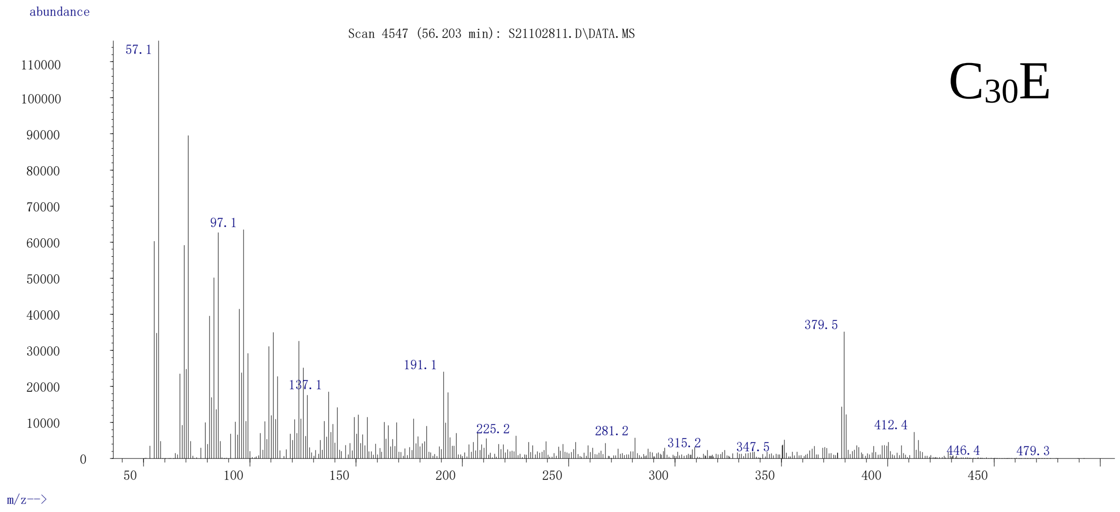

Figure 2. Representative mass spectrum of $C_{30}E$ (sample X762, retention time = 56.203 min).

The mass spectrum of $C_{30}E$ in this mature source rock sample was carefully examined. The identification is confidently based on the following key features:

- Molecular ion at m/z 412: Confirms the $C_{30}H_{52}$ molecular formula.

- Key Diagnostic Ion at m/z 191: While the nominal base peak is at m/z 57 (likely due to minor background aliphatic interference), the m/z 191 peak is the most abundant and characteristic fragment ion for the hopanoid skeleton in the diagnostic higher mass range.

- Absence of Key Tm Interference: Critically, the spectrum does not show the major diagnostic ion for 17α(H)-22,29,30-trisnorhopane (Tm) at m/z 370. The prominent high-mass fragment is observed at m/z 379, ruling out significant co-elution with Tm.

- Retention Time: The compound elutes at the precise retention time expected for C_{30} neohopane ($C_{30}E$) based on established elution patterns.

While the diagnostic m/z 287 fragment for $C_{30}E$ is of low intensity in this particular spectrum, the identification is considered robust based on the combination of its correct relative retention time, the prominent m/z 191 fragment, and the clear absence of the primary diagnostic peak for Tm (m/z 370). This multi-faceted approach confirms the identity of the compound as $C_{30}E$.

C. Comparison with Literature Spectra

The observed fragment ion distributions of both $C_{30}E$ and $C_{30}D$ were compared with those described in the literature to ensure accurate identification:

**Compound**	**Diagnostic Ions (m/z)**	**Literature Source**
$C_{30}D$	412, 191, 231, 397	Xiao et al., 2019; Farrimond & Telnaes,1996
$C_{30}E$	412, 191, (287)*	Xiao et al., 2019
Tm	412, 191, 370	Common in mature oils (Peters et al., 2005)

Note: The m/z 287 ion was of low intensity in the analyzed sample, but identification of $C_{30}E$ was confirmed by the absence of the m/z 370 ion characteristic of Tm.

References

1. Xiao, H., Li, M., Zhang, S., et al. (2019). Identification, distribution and geochemical significance of four rearranged hopane series in crude oil. Organic Geochemistry, 138, 103929.

2. Farrimond, Paul , and Nils TelnÆS. "Three series of rearranged hopanes in Toarcian sediments (northern Italy)." Organic Geochemistry 25.3-4(1996):165-177.

3. Moldowan, J.M., Fago, F.J., et al. (1991). Application of biological markers in petroleum exploration. ACS Symposium Series, 450, 89-114.

4. Peters, K. E., Walters, C. C., & Moldowan, J. M. (2005). The biomarker guide (2nd ed., Vol. 2). Cambridge University Press.
